# Editorial: Moving Beyond Non-informative Prior Distributions: Achieving the Full Potential of Bayesian Methods for Psychological Research

**DOI:** 10.3389/fpsyg.2021.809719

**Published:** 2021-12-09

**Authors:** Christoph Koenig, Sarah Depaoli, Haiyan Liu, Rens van de Schoot

**Affiliations:** ^1^Department of Educational Psychology, Goethe University Frankfurt am Main, Frankfurt, Germany; ^2^Department of Psychological Sciences, University of California, Merced, Merced, CA, United States; ^3^Department of Methodology and Statistics, Utrecht University, Utrecht, Netherlands

**Keywords:** Bayesian statistics, Bayesian modeling and inference, prior distributions, informative prior distributions, cumulative science, psychological research

Over the last two decades, Bayesian statistics have been established as an alternative to the well-known frequentist approaches primarily based on maximum likelihood (ML) estimation (van de Schoot et al., 2017; Koenig and van de Schoot, 2018). With the possibility of incorporating background knowledge into new analyses, Bayesian methods can potentially transform psychological research into a truly cumulative scientific discipline. However, the primary tool to achieve this, namely informative prior distributions, remains a seemingly elusive concept, especially for novice users. Reasons include, but are not limited to, the frequent criticism regarding their alleged subjective nature and a lack of knowledge about methods to formalize background knowledge (Goldstein, 2006; Vanpaemel, 2011). These two aspects are the primary point of departure for the twelve articles in this Special Issue.

The first set of two articles provides interested readers with means to **comprehend** the nature and potential impact of prior distributions in general. As Depaoli et al. (p. 3) state, “Understanding the impact of priors, and then making subsequent decisions about these priors, is perhaps the trickiest element of implementing Bayesian methods.” Consequently, their tutorial paper presents an interactive Shiny app that enables novice and experienced users of Bayesian statistics to investigate and determine the impact of their specified prior distributions on model results. Arts et al. examine the impact of different prior distribution specifications for the variance parameter in a Bayesian approximate measurement invariance with alignment optimization (e.g., van de Schoot et al., 2013). The authors illustrate visually how the prior specification for the variance parameter affects the rank ordering of 30 countries in a large-scale assessment of the latent construct” willingness to sacrifice the environment.” Visualizing different outcomes aids in understanding the effect of various prior specifications on model results.

The second set of articles aims to **convince** interested readers of the benefits and advantages of weakly and fully informative prior distributions compared to their non-informative counterparts and frequentist ML estimation. The five articles illustrate these benefits across a wide range of statistical models, with a particular focus on small-sample situations. Tong and Ke show the benefits and advantages of using weakly and fully informative priors for the precision parameter in Bayesian non-parametric growth curve models. Their simulation demonstrates that using weakly or fully informative priors aids model convergence and the accuracy of the precision parameter of the Dirichlet process. This conclusion is essential, as previous research showed that the precision parameter is crucial for obtaining good results.

Similarly, Zyphur et al. show that using weakly or fully informative priors also aids model convergence and parameter accuracy for cross-lagged panel models. They concluded that using such priors increases model parsimony, estimate stability, and thus the general trustworthiness of results, compared to results obtained with ML estimation. When dealing with small samples, the role of Bayesian prior distributions becomes even more crucial for model convergence and parameter accuracy. Smid and Winter present a tutorial discussing the dangers and pitfalls of using default priors implemented in software for Bayesian structural equation models. They introduce an interactive Shiny app, where users can investigate the impact of various priors on model estimates, depending on sample size. Lüdtke et al. examine the stability of estimates across different Bayesian estimators in small-sample confirmatory factor analysis. The results show that estimates based on the posterior mean (EAP) produced more accurate estimates. Parameter estimates can be further stabilized using the four-parameter beta distribution for loadings and factor correlations (e.g., Merkle and Rosseel, 2018). The benefits of using this prior distribution in the weakly informative specification are present even when prior distributions are mildly misspecified. Another specification of weakly informative priors is illustrated in Zitzmann et al. In the context of multilevel latent variable models, they describe two strategies (direct and indirect; Zitzmann et al., 2015) to specify weakly informative priors for the group-level slope parameter. Their simulation results show that introducing additional information via these priors stabilizes the model and provides more accurate parameter estimates in small-sample situations.

Finally, the third set of articles focuses on different approaches to formalize background knowledge objectively. The ultimate aim is to build **confidence** in the specification and use of informative prior distributions. In this regard, Veen et al. focus on expert knowledge for specifying informative prior distributions. In their paper, they illustrate how the five-step method (Veen et al., 2017) is used for prior elicitation for the parameters of a latent growth curve model. They show how to aggregate expert knowledge and specify appropriate densities to be used in a Bayesian analysis. Moreover, they compare the prior densities with posterior densities from traditionally collected data and guide how to set up procedures for appropriate expert elicitation.

Van de Schoot et al. provide another example of eliciting expert knowledge and using it to specify informative prior distributions. They also use lesser-known Bayesian methods, such as tests for prior-data conflicts (Box, 1980), a scoring algorithm to incentivize truthful responses (John et al., 2012), and Bayes factors for replication success (Verhagen and Wagenmakers, 2014), to investigate the prevalence of questionable research practices among Dutch and Belgian early career researchers. These articles are complemented by three illustrations focusing on more quantitative ways to formalize background knowledge. In this regard, Tran et al. focus on formalizing background knowledge with systematic parameter reviews. These reviews consist of a systematic literature search for studies containing estimates of relevant model parameters and necessary transformations to make the parameter comparable across studies. They illustrate how to specify informative prior distributions based on these synthesized parameter estimates in the context of the Diffusion Decision Model (DDM; Ratcliff and McKoon, 2008). The two remaining studies extend this approach and illustrate ways to consider the similarity of the available background knowledge and demonstrate how to apply the necessary weighting of the contributions of the individual studies to the informative prior distribution. In this regard, Schulz et al. implement a distribution-based approach. In the context of mother-adolescent interaction behavior, they illustrate three methods for pooling results from previously conducted studies to specify informative prior distributions. Moreover, they show how to use expert knowledge to weigh the contribution of each previously conducted study and how to use these weights in a power prior approach (Carvalho and Ibrahim, 2021).

Lastly, Koenig illustrates how to specify informative prior distributions using random-effects meta-analytic models. In the context of Bayesian multiple regression models, they present a novel method based on propensity-score and mixed-effects meta-analytic approaches (Tipton, 2014; Cheung, 2015) for quantifying the similarity of background knowledge. Moreover, they illustrate how to use this similarity measure to specify similarity-weighted informative prior distributions, an evidence-based informative prior also based on the power prior concept (Kaplan and Depaoli, 2013; Ibrahim et al., 2015).

To enhance reproducibility, crucial for Bayesian papers with informative priors (van de Schoot et al., 2021), each article in this Special Issue is accompanied by comprehensive supplementary material, including annotated code, which provides researchers with the means to apply the models and methods directly to their Bayesian analyses. In conclusion, we hope that this Special Issue enables novice and more experienced Bayesian researchers to move beyond non-informative prior distributions and unlock the full potential of Bayesian methods for psychological research.

## Author Contributions

All authors listed have made a substantial, direct, and intellectual contribution to the work and approved it for publication.

## Conflict of Interest

The authors declare that the research was conducted in the absence of any commercial or financial relationships that could be construed as a potential conflict of interest.

## Publisher's Note

All claims expressed in this article are solely those of the authors and do not necessarily represent those of their affiliated organizations, or those of the publisher, the editors and the reviewers. Any product that may be evaluated in this article, or claim that may be made by its manufacturer, is not guaranteed or endorsed by the publisher.
